# Multisystem Involvement in a Pediatric Patient With Hypermobile Ehlers-Danlos Syndrome: A Case Report of the Diagnostic Complexity and Management Challenges

**DOI:** 10.7759/cureus.62083

**Published:** 2024-06-10

**Authors:** Michael J Cosare, Alexandra G Korkmaz, Vladimir Valencia, Lauren M Toledo, Madhura Butala

**Affiliations:** 1 Department of Medicine, Lake Erie College of Osteopathic Medicine, Bradenton, USA; 2 Department of Pediatrics, Ascension St. Vincent's, Jacksonville, USA

**Keywords:** hypermobility, urticaria, mastocytosis, microscopic hematuria, col9a2 gene, hypermobile ehlers-danlos syndrome

## Abstract

Ehlers-Danlos syndrome (EDS) is a collection of genetic disorders caused by abnormalities in collagen and typified by hyperflexible joints, hyperextensible skin, and a tendency for easy bruising and tissue injuries. Hypermobile Ehlers-Danlos syndrome (hEDS), the most common subtype, presents a diagnostic challenge due to the lack of specific genetic markers. This case report describes a 13-year-old girl with hEDS, presenting with hypermobility, thoracolumbar scoliosis, constipation, glucosuria, microscopic hematuria, urticaria, and intermittent episodes of bilateral hand and feet swelling. Genetic testing revealed a variant of uncertain significance in the COL9A2 gene. An echocardiogram showed a mildly dilated aortic root. The complexity of her presentation underscores the challenges in diagnosing and managing hEDS with multisystem involvement.

## Introduction

Ehlers-Danlos syndrome (EDS) is a group of hereditary connective tissue disorders characterized by a wide range of clinical manifestations, including joint hypermobility, skin hyperextensibility, and tissue fragility. The pathophysiology of EDS is primarily related to defects in collagen synthesis and structure, as well as abnormalities in other extracellular matrix components. These defects lead to the characteristic symptoms of the syndrome, which can range from mild skin and joint issues to more severe complications such as arterial rupture and organ prolapse [1].

The syndrome encompasses a heterogeneous group of conditions, with 13 subtypes currently recognized, each having distinct genetic and clinical features. The prevalence of EDS varies depending on the subtype, with the hypermobile Ehlers-Danlos syndrome (hEDS) being the most common, estimated to affect one in 5,000 to one in 20,000 individuals [2]. Diagnosis of EDS is based on clinical criteria, family history, and, in some cases, genetic testing. The 2017 International Classification for Ehlers-Danlos Syndromes reports that of the 13 subtypes of EDS, diagnosis of hEDS remains challenging due to the lack of a specific genetic marker. The genetic basis of hEDS is not yet fully understood [1]. Management of EDS is primarily supportive and includes physical therapy, pain management, and surveillance for potential complications. Surgical interventions may be necessary for specific complications but carry a higher risk due to tissue fragility [3].

EDS, particularly the hypermobile type, has also been shown to have an increased prevalence of idiopathic urticaria relative to the random co-occurrence of the two disorders in the general population. A comprehensive meta-analysis of over two million charts by Szalewski and Davis showed that there is a statistically significant correlation between idiopathic urticaria and individuals with EDS compared to the general population. The urticaria in these cases has been implicated to be due to mast cell dysregulation. Mast cells play a significant role in homeostatic surveillance through recognition and response to different pathogens and injury via certain chemical mediators. Once recruited into connected tissues, resident mast cell progenitors differentiate with the influence of signals from the environment. A large number of mast cells reside within connective tissues including the skin, urogenital, respiratory, and gastrointestinal tracts. Immunohistochemistry analysis has identified an increased number of chymase-positive mast cells in patients with connective tissue disorders such as EDS [4].

In this case report, we present a patient with a complicated subtype of EDS, illustrating the challenges in diagnosis and management, and highlighting the importance of a comprehensive and multidisciplinary approach to care.

## Case presentation

A 13-year-old female presented with concerns of hypermobility since the age of 10. At the time, she was fairly active in sports. She reported stiffness and pain with activities in which orthopedic evaluation revealed thoracolumbar scoliosis. Additionally, she experienced constipation for several years and eventually began treatment with Miralax. The treatment had briefly managed her symptoms, but the patient reported constant flares of constipation.

Since experiencing back pain, the patient would additionally have intermittent episodes of bilateral hand and foot swelling. These unprovoked episodes would occur two to three times per month and lasted approximately one to two hours in duration. The patient reported a flare that resulted in back pain, inability to walk, and a rash, which was treated with high-dose corticosteroids. Sonogram of bilateral knee joints was negative for evidence of synovitis, tendinosis, and enthesitis. The rash was accompanied by urticaria, which recurred on a daily basis without any particular trigger, with episodes lasting three to four days. These episodes were described as “tingly” and were most prominent in the hands and lips. No bruising or scarring was noted. She followed up with an allergy and immunology physician for the management of her urticaria and angioedema. Her current management consisted of cetirizine 20 mg twice daily, with plans to initiate omalizumab if her symptoms remained uncontrolled.

She was eventually seen for an endocrinology consultation due to a family history of type 1 diabetes and the presence of glucosuria. She was diagnosed with benign essential microscopic hematuria with no sign of renal dysfunction or nephrolithiasis. She was also shown to have consistent neutropenia, which was suggested to be ethnic neutropenia. Alport syndrome and familial hematuria were ruled out at that time.

Nephrology and rheumatology were constantly followed during this time for microhematuria and glucosuria. Additional symptoms included frequent urination, back pain, and flank pain. Her workup included renal ultrasound and voiding cystourethrogram, which both were negative for any disease processes. Lab workup during that time demonstrated normal renal function without proteinuria. The low-titer positive antinuclear antibody (ANA) was thought to be insignificant at the time given the absence of underlying autoimmune processes.

**Figure 1:**
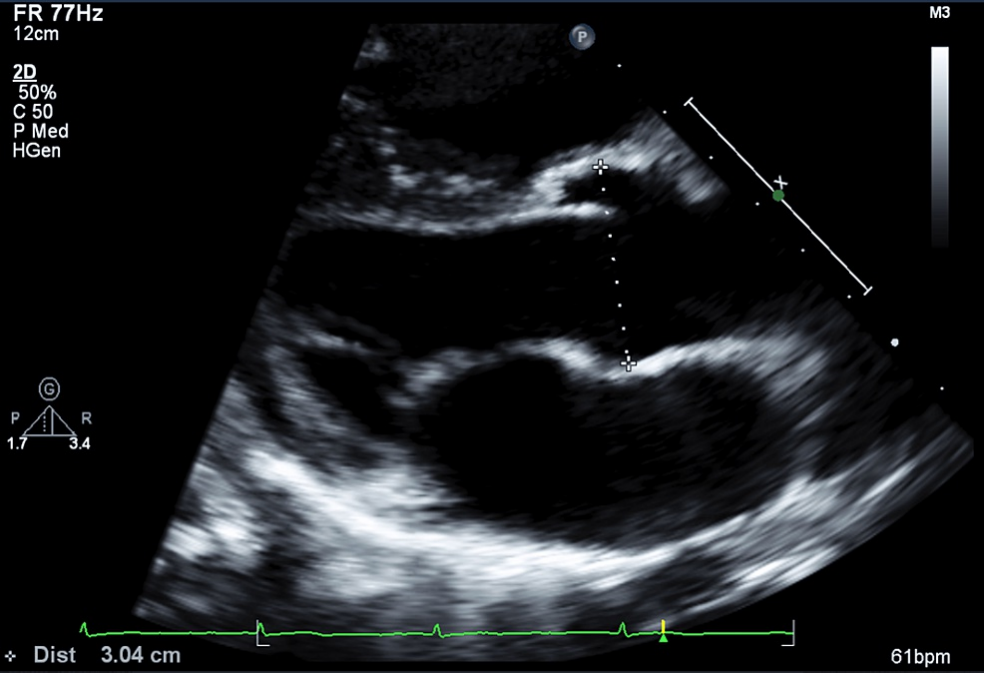
Mildly dilated aortic root in a pediatric patient with hypermobile Ehlers-Danlos syndrome

Moreover, cardiology consultation and echocardiography revealed a mildly dilated aortic root with a systolic aortic root dimension of 3.04 cm (Figure 1). Otherwise, cardiac valves, atria, ventricles, and other cardiac structures were found to be of normal structure and function. Following the diagnosis of the mildly dilated aortic root, she met the criteria for hEDS and was a candidate for further genetic testing.

Genetic testing revealed that she was heterozygous for COL9A2 with variant c.7C>A (p.Ala3Thr). The testing revealed that this is a variant of uncertain significance, but is known to be associated with multiple epiphyseal dysplasia (MED) and Stickler syndrome. In this case, the presence of a COL9A2 gene mutation raised the possibility of an association with MED and Stickler syndrome, although the patient's presentation did not include all typical features of these syndromes.

After years of clinical workup, the patient was diagnosed with hEDS on the basis of her clinical presentation and genetic findings. This diagnosis was supported by her history of joint hypermobility, skin hyperextensibility, and the absence of other definitive genetic markers for alternative diagnoses. The management of hEDS is primarily supportive, focusing on symptom relief, physical therapy, and surveillance for potential complications. In this patient's case, a multidisciplinary approach involving genetics, rheumatology, nephrology, urology, cardiology, allergy, and orthopedics was essential for comprehensive care and management of her complex presentation.

## Discussion

In patients with hEDS, there is a notable association with mast cell activation disease (MCAD), which includes conditions such as urticaria and mastocytosis. These conditions are characterized by an overactive or abnormal response of mast cells, leading to symptoms such as itching, flushing, and hives. The connection between hEDS and mast cell disorders is thought to be related to the underlying dysregulation of connective tissue in hEDS, which may impact mast cell behavior or activation thresholds. However, the exact mechanisms linking these conditions are still under investigation. The association with these conditions may be explained by the persistent chronic inflammation contributing to the disruption of connective tissue. Mast cell activation has been shown to play a role in the disruption of connective tissue integrity through the activity of certain mediators, including tryptase and histamine, resulting in MCADs. These mediators have been postulated to be the possible connection between hEDS, as they were found to promote the proliferation of fibroblasts and, therefore, the production of collagen [4].

The patient experienced multiple symptoms presumed related to aberrant mast cell activation. Her episodes of unprovoked urticaria and angioedema are likely related, as mast cells are among the most commonly implicated inflammatory cells in urticaria [1]. Proinflammatory cytokines, particularly IL-33, promote the adhesion, maturation, and degranulation of mast cells in urticaria. Additionally, she described neuropathic-type symptoms at the fingertips and lips. Mast cells are located in the epineurium, perineurium, and endoneurium of peripheral nerves. Ligament and capsular laxity have been postulated to result in abnormal pressure on peripheral nerves leading to these symptoms [5]. However, future studies on the establishment of the association between mast cell-related peripheral neuropathies and hEDS are warranted.

In addition, our patient’s genomic workup was revealed to be heterozygous for the COL9A2 gene with variant c.7C>A (p.Ala3Thr). This is a variant gene with unknown clinical significance. The COL9A2 gene, however, has been implicated in two main clinical syndromes: MED and Stickler syndrome. Both conditions are to be considered in this patient based on presentation, particularly the joint pain and stiffness she was experiencing.

MED is an autosomal dominant condition that presents typically with pain and weight-bearing joints in early childhood. Many children experience pain if fatigue occurs with long-distance walking or running or after periods of intense exercise. Once these individuals grow into adulthood, their height typically falls in the lower range of normal or may be considered mildly shortened. Patients may also additionally have shortening of limbs and deformities along with early onset manifestations of osteoarthritis. Radiographic findings may also include delayed ossification of the epiphysis or shortening of tubular bones. Genes that are typically associated with this condition include COL9A1, COL9A2, COL9A3, cartilage oligomeric matrix protein (COMP), and matrilin-3 (MATN3). Typical management and treatment include pain management, physiotherapy, and, if necessary later in life, total joint arthroplasty [6].

Stickler syndrome is a connective tissue disorder that can have variable presentation but is primarily defined by having ocular findings, hearing loss, underdevelopment of facial features, and variable skeletal manifestations. Stickler syndrome can be inherited in either an autosomal dominant or autosomal recessive pattern. Strong clinical suspicion should be had for Stickler syndrome when a patient presents with a combination of the aforementioned features on clinical presentation, or if imaging findings are suggestive of hip or knee involvement, or evidence of early onset degenerative joint disease. Genetic mutations seen in COL11A1, COL11A2, COL9A1, COL9A2, and COL9A3 are of importance when it comes to diagnosis. The patient in this case was heterozygous for the COL9A2 gene with the variant c.7C>A (p.Ala3Thr) [7].

In all, the patient's presentation highlights the diagnostic challenges associated with hEDS, particularly when accompanied by multisystem involvement. The differential diagnosis includes conditions such as MED and Stickler syndrome, which share overlapping features with hEDS. Genetic testing, including exome sequencing or multi-gene panels targeting specific genes, is crucial for diagnosis. In this case, the presence of a specific COL9A2 gene mutation raised the possibility of an association with Stickler syndrome, although the patient's presentation did not include all typical features of the syndrome.

## Conclusions

The diagnosis of hEDS, in this case, was challenging due to the overlap of symptoms with other conditions and the variant of uncertain significance in the COL9A2 gene. The differential diagnosis included MED and Stickler syndrome, both of which are associated with COL9A2 mutations. However, the absence of characteristic radiographic findings and the patient's clinical presentation led to the diagnosis of hEDS.

Management of hEDS is multidisciplinary, focusing on symptom relief and prevention of complications. In this case, a team of specialists in genetics, rheumatology, nephrology, urology, cardiology, allergy, and orthopedics were involved in the patient's care. Regular follow-up is crucial for monitoring the condition and adjusting the management plan as needed. This case report underscores the complexity of diagnosing and managing hEDS with multisystem involvement. A thorough clinical evaluation and genetic testing are essential for accurate diagnosis and appropriate management.
